# Integrated bioinformatic analysis of the shared molecular mechanisms between ANCA-associated vasculitis and atherosclerosis

**DOI:** 10.1186/s13075-024-03448-w

**Published:** 2024-12-19

**Authors:** Xun Hu, Inmaculada Xu Lou, Qilan Chen

**Affiliations:** 1 Hangzhou Hospital of Traditional Chinese Medicine Affiliated to Zhejiang Chinese Medical University, Hangzhou, Zhejiang 310053 China; 2 Department of Cardiology, Hangzhou Hospital of Traditional Chinese Medicine, Hangzhou, Zhejiang 310025 China

**Keywords:** ANCA-associated vasculitis, Atherosclerosis, Bioinformatics, Differentially expressed genes, Hub genes, Immune cell infiltration

## Abstract

**Background and objective:**

Accumulated evidence supports the tendency of antineutrophil cytoplasmic antibody (ANCA)-associated vasculitis(AAV) to coexist with atherosclerosis (AS). However, the common etiology of these two diseases remains unclear. This study aims to explore the mechanisms underlying the concurrent occurrence of ANCA and AS.

**Methods:**

Microarray data of AAV and AS were examined in a comprehensive gene expression database. Weighted gene co-expression network analysis (WGCNA) and differential gene expression analysis (GEO2R) were performed to identify common genes between AAV and AS. Based on the co-expressed genes, functional enrichment analysis, protein-protein interaction (PPI) network analysis, and identification of hub genes (HGs) were conducted. Subsequently, co-expression analysis of HGs was performed, and their expression and diagnostic value were validated. We further explored immune cell infiltration and analyzed the correlation between HGs and infiltrating immune cells. Finally, the reliability of the selected pathways was verified.

**Results:**

The results of the common gene analysis suggest that immune and inflammatory responses may be common features in the pathophysiology of AAV and AS. Through the interaction of different analysis results, we confirmed five HGs (CYBB, FCER1G, TYROBP, IL10RA, CSF1R). The CytoHubba plugin and HG validation demonstrated the reliability of the selected five HGs. Co-expression network analysis revealed that these five HGs could influence monocyte migration. Analysis of immune cell infiltration showed that monocytes in ANCA and M0 macrophages in AS constituted a higher proportion of all infiltrating immune cells, with significant differences in infiltration. We also found significant positive correlations between CYBB, FCER1G, TYROBP, IL10RA, CSF1R, and monocytes/M0 macrophages in AAV, as well as between CYBB, FCER1G, TYROBP, IL10RA, CSF1R, and M0 macrophages in AS.

**Conclusion:**

These five HGs can promote monocyte differentiation into macrophages, leading to the concurrent occurrence of AAV and AS. Our study provides insights into the mechanisms underlying the coexistence of AAV and AS.

## Introduction

The prognosis of patients with ANCA-associated vasculitis (AAV) has improved in the past few decades, but the overall mortality rate remains higher than the general population, with cardiovascular events being the most common cause of death. Massicotte-Azarniouch et al. conducted a large retrospective cohort study to assess cardiovascular disease (CVD) outcomes in AAV patients compared to non-AAV individuals. Their research demonstrated a significant increase in cardiovascular risk among AAV patients, highlighting them as a high-risk group for CVD. AAV is characterized by inflammation, and atherosclerosis (AS) is a chronic inflammatory disease of the arterial intima. The inflammatory nature of AAV is believed to be a major factor accelerating atherosclerosis in these patients. Systemic inflammation and immune abnormalities contribute to the accelerated atherosclerosis, independent of classical risk factors. Endothelial dysfunction, a recognized CVD risk factor, has been shown to exist in AAV and is unrelated to disease activity or kidney involvement [1].

Although AAV is considered a risk factor for AS, the shared pathogenic mechanisms between these two diseases remain unclear. Potential underlying mechanisms for the accelerated atherosclerosis in AAV include the increase of different mediators, such as metalloproteinases, VEGF, and PDGF, in vasculitis, leading to intimal proliferation and luminal narrowing. Inflammatory cytokines also impact coagulation via thrombomodulin-C [2]. Some pro-inflammatory cytokines, such as CRP, enhance atherosclerosis formation by increasing adhesion molecule expression, cell recruitment, smooth muscle cell stimulation, and macrophage apoptosis [3]. Other potential mechanisms involve the infiltration of activated inflammatory cells within the arterial wall. The increase in inflammation and autoantibody production in AAV and macrophage activation in AS are related to the formation of neutrophil extracellular traps [4]. Specific antiphospholipid antibodies and lupus anticoagulant are commonly present in AAV patients. These autoantibodies in AAV induce immune complex formation, which promotes disease pathogenesis by fixing complement, binding to neutrophil Fcg receptors, and activating neutrophils [5].

However, the representation of their sample was limited and there was a lack of studies integrating genetic data from public databases on AAV and AS. Our aim is to explore the shared pathogenic mechanisms between AAV and AS based on their common transcriptional features and provide new insights into the biological mechanisms of both diseases.

## Materials and methods

### Data source

The microarray datasets were downloaded from the Gene Expression Omnibus (GEO) database (http://www.ncbi.nlm.nih.gov/geo/), which contains a large collection of high-throughput sequencing and expression microarray data. Relevant gene expression datasets were searched using the keywords “ANCA” and “atherosclerosis,” excluding non-human samples. Finally, the datasets with the accession numbers GSE108113, GSE104948, GSE104954, GSE100927, GSE43292, and GSE28829 were downloaded from the GEO database.

### Identification of common genes through DEG Analysis

Differential expression genes (DEGs) in the GSE108113 and GSE100927 datasets were determined by comparing the gene expression profiles between the disease group and the control group using “limma” package in R (version 4.2.3). The normalizeBetweenArrays function in the limma package is used for normalization, and the removeBatchEffect function is used to remove batch effects. |logFC| > 1 and adjusted p-value < 0.05 were considered to indicate statistical significance. A Venn diagram was generated to obtain the common DEGs with the same trend.

### Weighted Gene Co-expression Network Analysis (WGCNA)

A systems biology approach called Weighted Gene Co-Expression Network Analysis (WGCNA) is used to analyze co-expressed gene modules with high biological significance and explore the relationship between gene networks and diseases. Therefore, we performed WGCNA analysis on the GSE104948 and GSE43292 datasets to obtain modules related to AAV and AS. NormalizeBetweenArrays and removeBatchEffect functions in the limma package are used to normalize and remove batch effects. Using the R language, genes with a median absolute deviation greater than 25% were selected before the analysis, and outliers were removed from hierarchical clustering analysis using the Hclust function. The appropriate soft-thresholding power β was calculated to achieve a scale-free topology with a criterion of R² > 0.85, and the minimum module gene size was set to 30. Finally, module eigengenes and the correlation between module eigengenes and clinical features were computed to obtain expression profiles of each module. Therefore, we focused on the modules with high correlation coefficients with clinical features and selected genes from these modules for further analysis.

### Analysis of Gene Modules through WGCNA Analysis

Using the Pearson correlation coefficient and the p-values of characteristic genes and disease features in each module, we determined the key modules associated with AAV and AS. Then, by utilizing the genes from the key modules that were positively correlated with AAV and AS, we obtained the common genes through a Venn diagram.

### Enrichment analysis

To analyze the biological functions and pathways of DEG genes, the “clusterProfiler” package in R software was used to perform enrichment analysis on Gene Ontology (GO) terms and Kyoto Encyclopedia of Genes and Genomes (KEGG) pathways. GO analysis includes three categories: cellular component analysis (CC), biological process analysis (BP), and molecular function analysis (MF).

### Hub gene selection and validation

The shared genes of AAV and AS were obtained by intersecting the common genes from CDEG and WGCNA. The protein-protein interaction (PPI) network was analyzed using the Search Tool for the Retrieval of Interacting Genes (STRING; http://string-db.org). Interactions with a combined score > 0.4 were selected, and the PPI network was constructed using Cytoscape software (version 3.10.0). Then, for the benefit of credibility, using the CytoHubba plugin in Cytoscape, seven algorithms were randomly selected, and the intersection results were used to identify hub genes. Additionally, the expression of hub genes was validated in GSE104954 and GSE28829. The comparison between the two datasets was performed using t-tests, and a p-value < 0.05 was considered significant.

### Assessment and correlation analysis of Immune Cell infiltration by HGs and IICs

We utilized the “CIBERSORT” R package algorithm in R language to analyze gene expression data from GSE104948 and GSE43292 (*p* < 0.05). Subsequently, we employed the “corrplot,” “pheatmap,” and “ggplot2” R packages to generate correlation heatmaps and box plots for IICs.

## Results

### GEO information

Six GEO datasets (namely, GSE108113, GSE104948, GSE104954, GSE100927, GSE43292, and GSE28829) were selected in all. Detailed information of these six datasets is shown in Table 1.

**Table 1:** GEO datasets

ID	GSE number	Platform	Samples	Disease	Tissue
1	GSE108113	GPL19983	15 patients and 11 controls	ANCA-associated vasculitis	kidney biopsy
2	GSE104948	GPL22945	22 patients and 18 controls	ANCA-associated vasculitis	kidney biopsy
3	GSE104954	GPL19983	21 patients and 18 controls	ANCA-associated vasculitis	kidney biopsy
4	GSE100927	GPL17077	69 patients and 35 controls	atherosclerosis	artery
5	GSE43292	GPL6244	32 patients and 32 controls	atherosclerosis	artery
6	GSE28829	GPL570	16 patients and 13 controls	atherosclerosis	artery

### Identification of differentially expressed genes (DEGs)

**Fig. 1:**
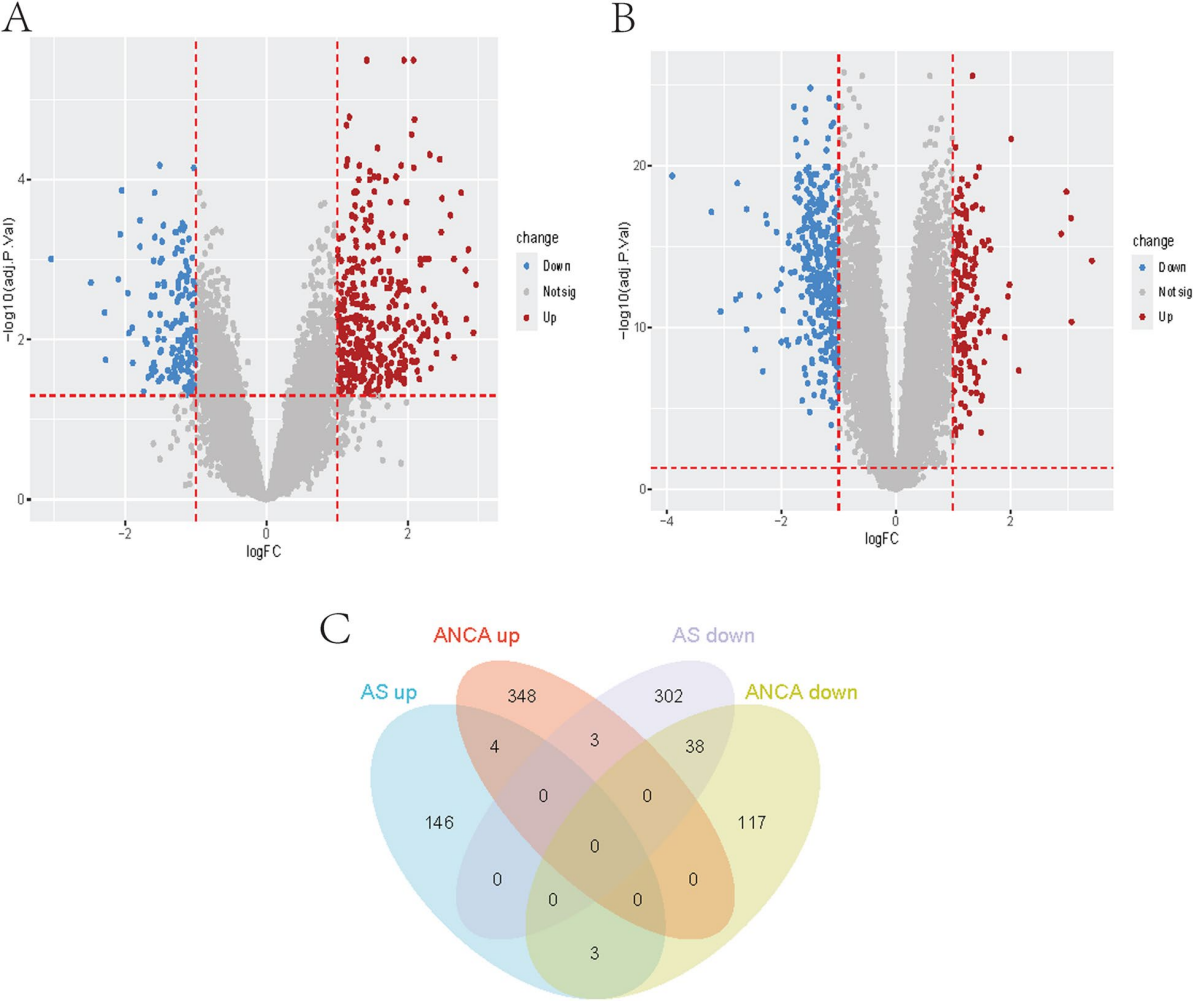
Volcano plot and Venn diagram. **A** Volcano plot of differentially expressed genes (DEGs) in GSE108113. **B** Volcano plot of differentially expressed genes (DEGs) in GSE100927. The up-regulated genes were marked with red, the down-regulated genes were marked with blue, and the genes with no significant changes were marked with gray. **C** Venn diagram of Same trend genes in GSE108113 and GSE100927

A total of 513 DEGs were obtained from the GSE108113 dataset, with 343 DEGs downregulated and 153 DEGs upregulated (Fig. 1A). Additionally, 513 DEGs were obtained from the GSE100927 dataset, with 158 DEGs downregulated and 355 DEGs upregulated (Fig. 1B). We performed an intersection analysis of the downregulated and upregulated DEGs from both datasets, resulting in 42 common DEGs with consistent expression trends, referred to as Common Differentially Expressed Genes (CDEGs) (Fig. 1C).

### Functional characterization analysis of DEGs

**Fig. 2:**
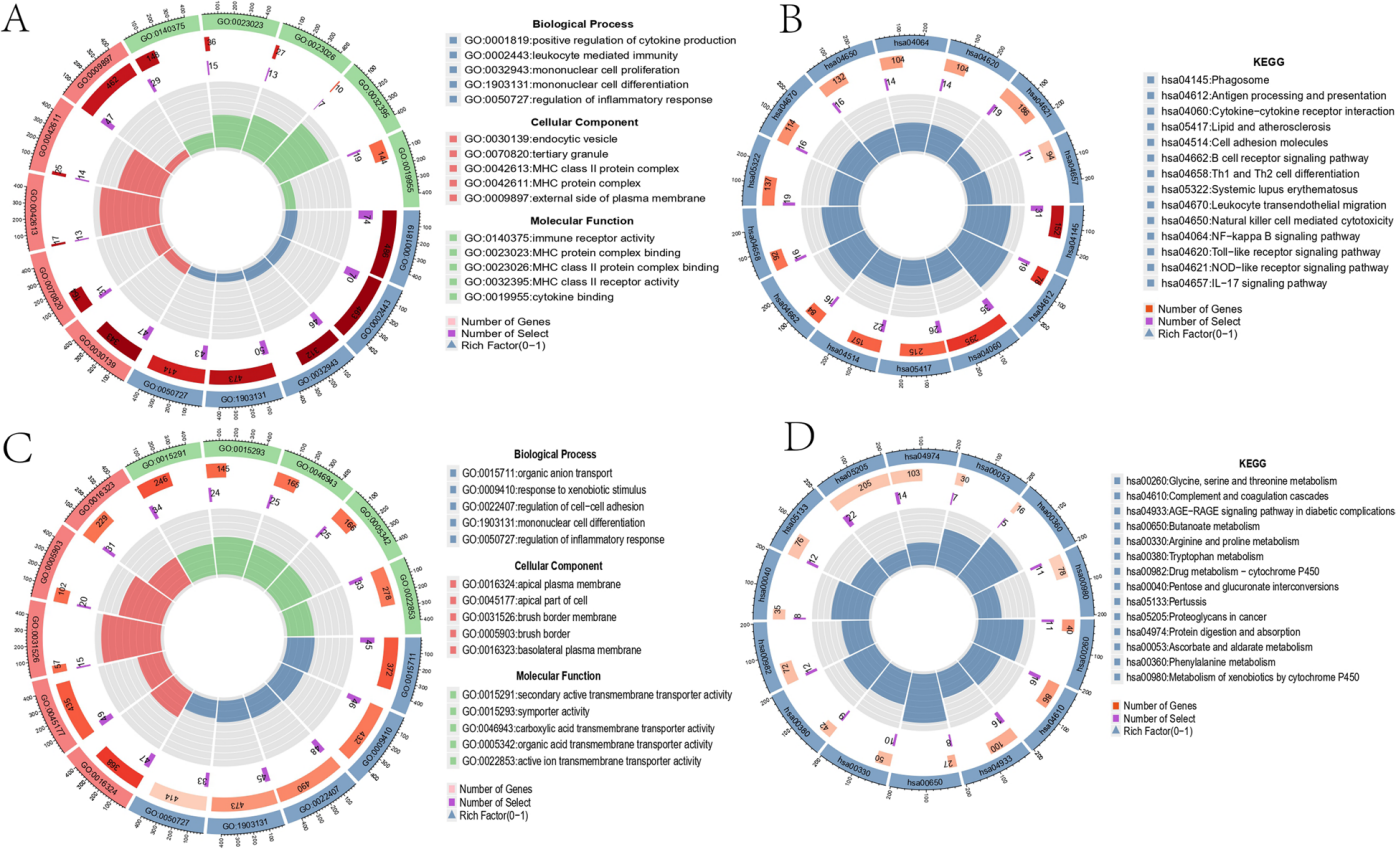
Analysis of functional characteristics of DEGs. **A**-**B** Bar chart of the DEGs in GSE108113 KEGG and GO functional enrichment analysis. **C**-**D** Bar chart of the DEGs in GSE100927 functional enrichment analysis

We performed gene ontology (GO) term enrichment analysis and Kyoto Encyclopedia of Genes and Genomes (KEGG) pathway analysis of the DEGs in GSE108113 and GSE100927 using the “clusterProfiler” package in R software. We found significant enrichment of organic anion transport, response to xenobiotic stimulus, regulation of cell-cell adhesion, mononuclear cell differentiation, and regulation of inflammatory response in AAV (Fig. 2A, B). Additionally, we observed significant enrichment of positive regulation of cytokine production, leukocyte mediated immunity, mononuclear cell proliferation, mononuclear cell differentiation, and regulation of inflammatory response in AS (Fig. 2C, D). Many immune and inflammation-related processes, such as mononuclear cell differentiation and regulation of inflammatory response, play important roles in AAV and AS.

### Co-expression modules of two diseases

**Fig. 3:**
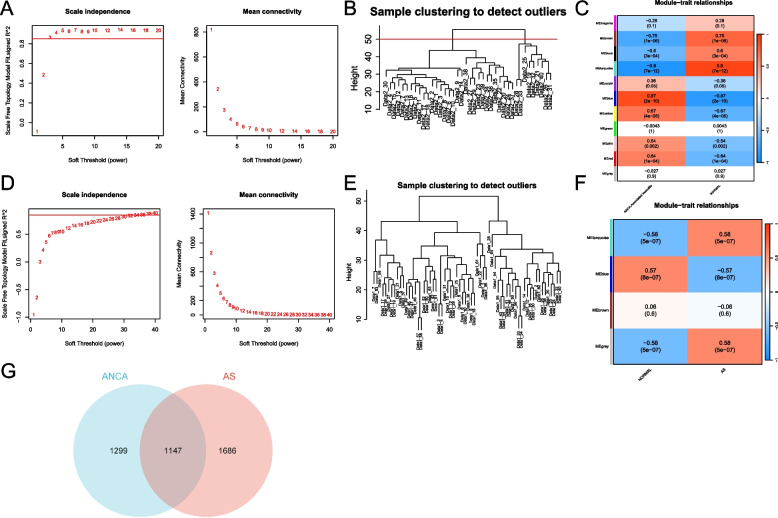
Weighted genes correlation network analysis (WGCNA) of GSE104948 and GSE43292 datasets. **A** Soft threshold analysis in AAV. **B** Clustering analysis in AAV. **C** Heatmap of the module-trait relationship in AAV. Each cell contains the corresponding correlation and *p* value. **D** Soft threshold analysis in atherosclerosis. **E** Clustering analysis in AS. **F** Heatmap of the module-trait relationship in atherosclerosis. Each cell contains the corresponding correlation and *p* value. **G** Venn diagram of common genes were identified from the positively correlated modules between AAV and AS

As shown in Fig. 3A, co-expression modules related to the AAV phenotype were obtained through WGCNA analysis of the GSE104948 dataset, and clustering analysis indicated the exclusion of 2 outlier samples (Fig. 3B). The soft threshold selection analysis indicated that the gene correlations were maximally consistent with a scale-free distribution when β = 3 (scale-free R^2 = 0.85). Subsequently, 9 modules were identified in the weighted gene co-expression network by merging modules with a module eigengene value greater than 0.5 and a minimum gene count set at 30. As shown in Fig. 3C, the MEbrown module (*r* = 0.75, *p* = 1e-06), MEblack module (*r* = 0.6, *p* = 3e-04), MEturquoise module (*r* = 0.9, *p* = 7e-12), MEblue module (*r* = 0.87, *p* = 2e-10), MEyellow module (*r* = 0.67, *p* = 4e-05), MEpink module (*r* = 0.54, *p* = 2e-03), and MEred module (*r* = 0.54, *p* = 2e-03) were highly correlated with AAV. A total of 2446 genes from these 7 modules were further analyzed.

Similarly, as shown in Fig. 3F, WGCNA analysis of the GSE43292 dataset yielded 4 gene modules, and clustering analysis does not exclude samples (Fig. 3E). Furthermore, as shown in Fig. 3F, a heatmap of module-trait relationships based on Spearman correlation coefficients was generated to assess the associations between each module and the diseases. Among these 4 modules, the “MEturquoise”, “MEblue” and “MEgrey” modules exhibited higher correlations with AS (MEturquoise module: *r* = 0.58, *p* = 5e-07; MEblue module: *r* = 0.57, *p* = 8e-07; MEgrey module: *r* = 0.58, *p* = 5e-07), totaling 2833 genes. Subsequently, as shown in Fig. 3G and 1147 common genes were identified from the 10 positively correlated modules between AAV and AS.

### Analysis of common gene targets

**Fig. 4:**
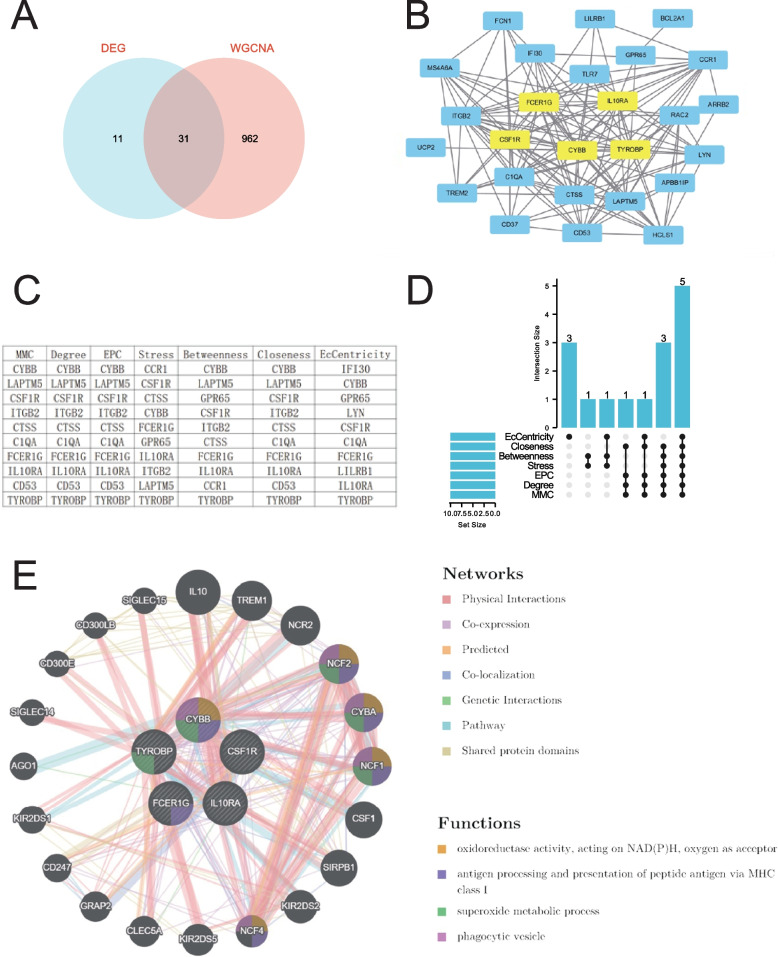
**A** Venn diagram of common genes between DEG and WGCNA. **B** Location of the five HGs in the PPI network. **C** The top 10 HGs as ranked in CytoHubba. **D** Up-Set diagram of the results screened by seven algorithms of CytoHubba. **E** Five HGs and their co-expression genes analyzed using GeneMANIA

To integrate the biological data reported, we visualized the intersection of DEGs and WGCNA by Venn diagram (Fig. 4A), resulting in 31 common genes. These common genes were imported into the STRING database, and one gene that failed to interact was excluded. The PPI (protein-protein interaction) network of the 31 common gene targets between AAV and AS was generated. We randomly utilized seven algorithms (MMC, Degree, EPC, Stress, Betweenness, Closeness, EcCentricity) from the CytoHubba plugin to identify hub genes (HGs). Figure 4C lists the top 10 genes selected by the seven algorithms. By intersecting the results of the seven algorithms, we identified five HGs (CYBB, FCER1G, TYROBP, IL10RA, and CSF1R) using the upSet plot (Fig. 4D). Figure 4B displays the positions of these five HGs in the PPI network.

To further explore the biological characteristics of the HGs, we constructed and analyzed a network using GeneMANIA in Fig. 4E, including the HGs and their co-expressed genes. The five HGs exhibited a complex PPI network composition: Physical Interactions (77.64%), Co-expression (8.01%), Predicted (5.37%), Co-localization (3.63%), Genetic Interactions (2.87%), Pathway (1.88%), and Shared protein domains (0.60%). The biological functions of the HGs were associated with oxidoreductase activity, acting on NAD(P)H, oxygen as acceptor, antigen processing and presentation of peptide antigen via MHC class I, superoxide metabolic process, and phagocytic vesicle.

### Validation of expression and diagnostic value of HGs

**Fig. 5:**
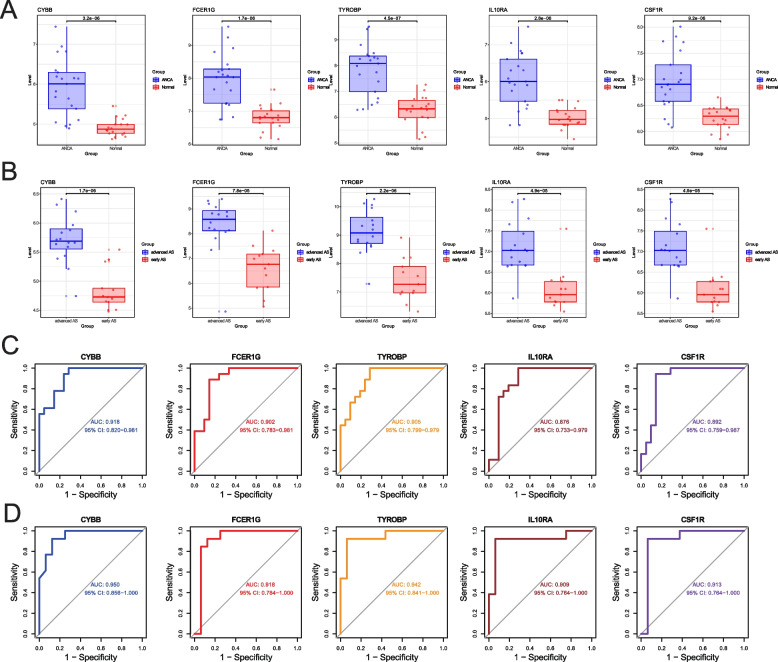
Validation of HGs expression and diagnostic value. **A** Expression of the five HGs verified in GSE104954. **B** Expression of the five HGs verified in GSE48829. **C** ROC curves of five HGs in GSE104954. **D** ROC curves of five HGs in GSE48829

To validate the reliability of the selected HGs, the expression of the five HGs was validated using the GSE104954 and GSE28829 datasets. The results showed that the expression levels of the five HGs were higher in AAV compared to the control samples (Fig. 5A), and the expression of these five HGs was also higher in AS plaques compared to the control samples (Fig. 5B). The receiver operating characteristic (ROC) curves of the five HGs are shown in Fig. 5C for AAV and Fig. 5D for AS, demonstrating good diagnostic value in both AAV and AS. This indicates that the selected five HGs are reliable.

### Results of Immune Cell Infiltration

**Fig. 6:**
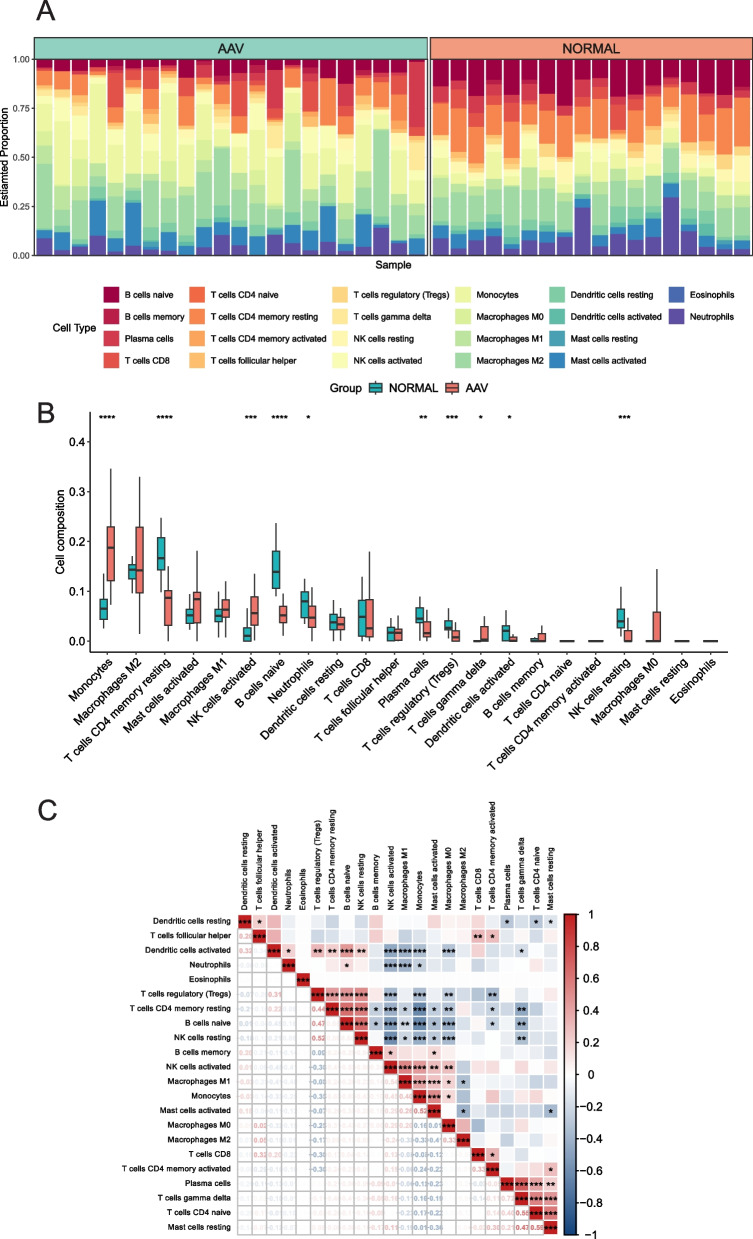
Landscape map of IICs in the ANCA-associated vasculitis and Normal groups. **A** The relative proportion of IICs in ANCA and Normal. **B** Box diagram showing the difference in immune cell infiltration between ANCA and Normal. (ANCA group shown in red, Normal group shown in green, *p* < 0.05 was considered statistically significant). **C** Correlation heatmap between IICs. Red represents positive correlation, blue represents negative correlation, and the number in the square represents correlation

Figure 6A shows the composition of immune cells in the AAV group and the control group. We found that the AAV group had significantly higher numbers of Monocytes, NK cells activated, and Macrophages M0 compared to the control group (*p* < 0.05), with Monocytes having the highest proportion among all immune and inflammatory cells (IICs). On the other hand, the numbers of T cells CD4 memory resting, B cells naive, Neutrophils, Plasma cells, T cells regulatory (Tregs), Dendritic cells activated, and NK cells resting were significantly lower in the AAV group compared to the control group (*p* < 0.05) (Fig. 6B). Additionally, we generated a heatmap of the correlations among the IICs (Fig. 6C). We observed negative correlations between Monocytes and Dendritic cells activated (*r* = −0.33), Tregs (*r* = −0.35), T cells CD4 memory resting (*r* = −0.63), B cells naive (*r* = −0.63), and NK cells resting (*r* = −0.43). Furthermore, Monocytes showed a positive correlation with Macrophages M1 (*r* = 0.4), NK cells activated (*r* = 0.45), Mast cells activated (*r* = 0.52) and Macrophages M0 (*r* = 0.16).

### Composition of Immune cells in AS Plaque tissue and Control Group

**Fig. 7:**
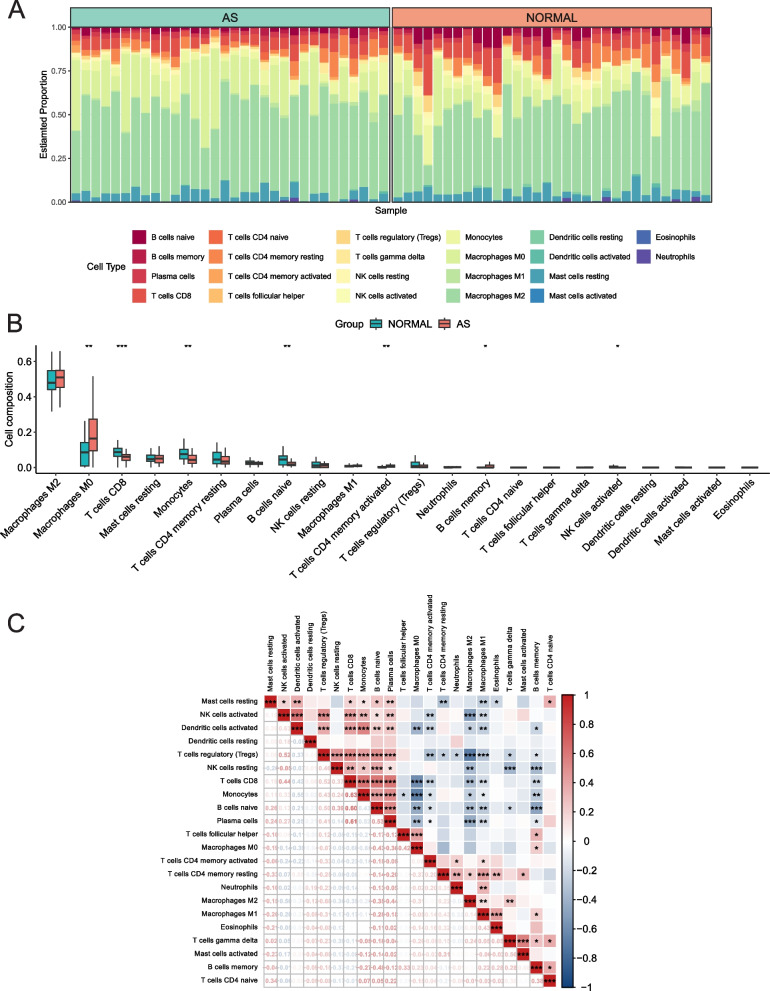
Landscape map of IICs in the Atherosclerosis and Normal groups. **A** The relative proportion of IICs in AS and Normal. **B** Box diagram showing the difference in immune cell infiltration between AS and Normal. (AS group shown in red, Normal group shown in green, *p* < 0.05 was considered statistically significant). **C** Correlation heatmap between IICs. Red represents positive correlation, blue represents negative correlation, and the number in the square represents correlation

The composition of immune cells in AS plaque tissue and the control group was shown. We found that Macrophages M0, T cells CD4 memory, and B cells memory activated were significantly higher in the AS plaque tissue compared to the control group, as shown in Fig. 7B (*p* < 0.05), with Macrophages M0 having a higher proportion among all IICs (Fig. 7A). On the other hand, T cells CD8, Monocytes, B cells naive, and NK cells activated were significantly lower in the AS plaque tissue compared to the control group (*p* < 0.05) (Fig. 7B). The heatmap of correlations among the IICs (Fig. 7C) showed that Macrophages M0 had a negative correlation with Dendritic cells activated (*r* = −0.39), T cells CD8 (*r* = −0.66), Monocytes (*r* = −0.65), B cells naive (*r* = −0.43), and Plasma cells (*r* = −0.36), while it had a positive correlation with T cells follicular helper (*r* = 0.42), and B cells memory (*r* = 0.23), with all correlations being significant at *p* < 0.05.

### Correlation analysis between HGs and IICs

**Fig. 8:**
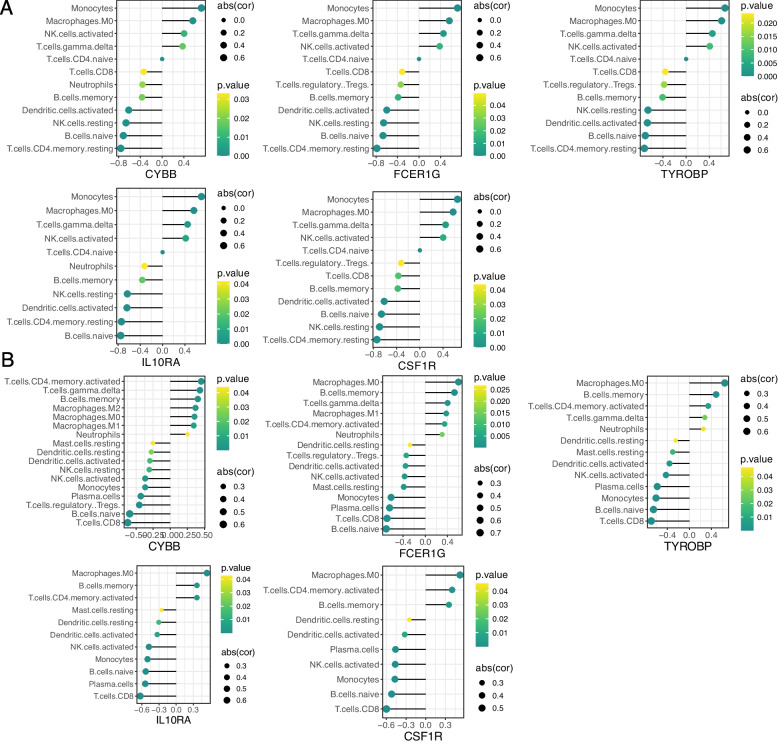
Correlation between five HGs and IICs. **A** Correlation between five HGs and IICs in AAV. **B** Correlation between five HGs and IICs in AS

We analyzed the correlation between the 5 HGs and IICs in AAV (Fig. 8A). We found significant positive correlations (*p* < 0.05) between CYBB, FCER1G, TYROBP, IL10RA, CSF1R, and Monocytes, Macrophages.M0, T cells gamma delta, NK cells activated. Additionally, we analyzed the correlation between the 5 HGs and IICs in AS (Fig. 8B). We observed significant positive correlations (*p* < 0.05) between the 5 HGs and Macrophages.M0, B.cells.memory, T.cells.CD4.memory.activated, and a significant positive correlation (*p* < 0.005) with Macrophages M0. Therefore, these 5 HGs may play important roles in the pathogenesis of AAV combined with AS by influencing immune cell infiltration. Monocytes and Macrophages M0 may be the targets of these 5 HGs.

## Discussion

The physiology of ANCA involves a complex interaction between ANCA antibodies, neutrophils, and other components of the immune system [6, 7]. ANCA antibodies are generated in response to specific antigens present in neutrophils and monocytes, such as proteinase 3 (PR3) [8] and myeloperoxidase (MPO) [9]. When ANCA antibodies bind to these antigens, they can activate neutrophils, leading to the release of enzymes and proinflammatory substances stored in their granules [10, 11]. This results in the production of reactive oxygen species (ROS) and a localized inflammatory response in the tissues. This inflammation can cause tissue damage and dysfunction of the involved organs, such as renal or pulmonary failure [12–18].

Atherosclerosis is a chronic inflammatory disease of the arteries, where the endothelium is damaged due to risk factors such as hypertension, smoking, high cholesterol, and diabetes. This allows LDL to enter and accumulate in the arterial wall, especially when it undergoes oxidation. The inflammatory response attracts inflammatory cells such as monocytes, which transform into foam cells by accumulating oxidized lipids. These cells release cytokines and growth factors that promote the formation of atherosclerotic plaques, consisting of lipids, calcium, and connective tissue. The plaques can become unstable and prone to rupture, leading to blood clot formation and arterial obstructions, resulting in severe cardiovascular events. Controlling risk factors is essential to prevent or delay the progression of this disease. Emerging evidence suggests that atherosclerosis is also an epigenetic disease involving the interaction of multiple epigenetic mechanisms [19–22].

ANCA-associated vasculitis and atherosclerosis, although distinct, may be related [23, 24]. Vasculitis affects blood vessels due to an autoimmune response, whereas atherosclerosis involves the accumulation of plaques in the arteries. However, patients with ANCA-associated vasculitis have a higher risk of atherosclerosis due to chronic inflammation, which triggers systemic inflammatory responses and endothelial damage, favoring the development of atherosclerosis [25–28]. The connection between ANCA and atherosclerosis is not fully established yet. ANCA are antibodies directed against components of neutrophils and are related to systemic vasculitis. Their specific role in atherosclerosis is the subject of investigation, but some studies suggest that they could be involved in inflammation and endothelial dysfunction, contributing to disease development [29]. Genetically, studies have been conducted to examine genetic variants associated with ANCA and atherosclerosis, identifying genetic polymorphisms related to the predisposition to ANCA-associated vasculitis and increased risk of atherosclerosis. Although not fully confirmed yet, these findings indicate the need for further research to understand the genetic basis and interrelation of these diseases [30]. Patients with ANCA-associated vasculitis show a higher frequency of subclinical markers of atherosclerosis and have an increased risk of cardiovascular events compared to the general population. Chronic inflammation, disease-induced endothelial dysfunction, and increased susceptibility to LDL oxidation are suggested to contribute to accelerated atherosclerosis in these patients [31–33]. A higher incidence of cardiovascular disease has also been observed in patients with systemic vasculitis, supporting the association between both conditions [34].

To the best of our knowledge, this is the first attempt to explore the comorbidity hypothesis between ANCA-associated vasculitis and atherosclerosis by integrating data from diverse public databases. The objective is to identify shared mechanisms underlying both ANCA-associated vasculitis and atherosclerosis. In our study, the results regarding ANCA-associated vasculitis showed an enrichment of organic anion transport, response to xenobiotic stimuli, regulation of cell-cell adhesion, mononuclear cell differentiation, and regulation of the inflammatory response. Monocytes, activated NK cells, and M0 macrophages were found to be activated, with monocytes standing out as the most abundant inflammatory cells. On the other hand, there was a lower activation of resting memory CD4 T cells, naive B cells, neutrophils, plasma cells, Tregs, dendritic cells activated and resting NK cells. Regarding the results on atherosclerosis, we observed an enrichment of positive regulation of cytokine production, leukocyte-mediated immunity, mononuclear cell proliferation, mononuclear cell differentiation, and regulation of the inflammatory response in AS. Elevated levels of M0 macrophages, T cells CD4 memory, and activated B cells memory were found, with M0 macrophages standing out as the most abundant immune and inflammatory cells. On the other hand, we observed a lower activation of CD8 T cells, monocytes, naive B cells, and NK cells.

Numerous diseases are associated with inflammation and/or the immune system, many of which are autoimmune disorders or chronic inflammatory conditions. Examples include rheumatoid arthritis, systemic lupus erythematosus (SLE), celiac disease, Crohn’s disease, ulcerative colitis, type 1 diabetes, allergies, asthma, autoinflammatory diseases (e.g., familial Mediterranean fever), vasculitis (e.g., granulomatosis with polyangiitis), Alzheimer’s disease, psoriasis, Sjögren’s syndrome, and multiple sclerosis. These conditions have diverse manifestations and may require treatments such as immunosuppressants, anti-inflammatories, immune response modifiers, and targeted therapies. Diagnosis and tailored management are crucial due to their varying inflammatory and immune mechanisms [35–40].

The expression of the genes CYBB, FCER1G, TYROBP, IL10RA, and CSF1R is increased in both ANCA-associated vasculitis and atherosclerosis. These findings suggest a potential relationship between both diseases through the immune system and inflammatory mechanisms, involving the regulation of leukocyte, lymphocyte, and mononuclear cell proliferation, antigen presentation, and the presence of ficolin-1-rich granules. Additionally, our study revealed a positive correlation between the mentioned genes (CYBB, FCER1G, TYROBP, IL10RA, CSF1R) and the activation of monocytes, M0 macrophages, activated NK cells, and gamma delta T cells. Monocytes and M0 macrophages are proposed as possible targets regulated by these five genes.

The CYBB gene encodes a subunit of NADPH oxidase, which is essential for the production of ROS [41]. In the case of AAV, excess ROS contributes to endothelial dysfunction, while in AS, it promotes LDL oxidation, thereby enhancing inflammation and the recruitment of macrophages to atherosclerotic plaques [42]. Additionally, FCER1G encodes a protein involved in immune signaling through Fc receptors and activates immune cells such as neutrophils and macrophages [43, 44]. In AAV, the activation of these immune cells may contribute to inflammation and vascular damage [45], whereas in AS, it exacerbates the immune response in arterial walls [46]. Also, TYROBP regulates the activation of immune cells and is critical for immune receptor signaling [47–49]. Its overexpression can promote the activation of macrophages and dendritic cells, increasing the risk of autoimmune inflammation and vascular damage. Furthermore, the activation of immune cells in the arterial wall may contribute to chronic inflammation in atherosclerotic plaques [50]. Moreover, IL10RA encodes a subunit of the receptor for IL-10. This anti-inflammatory interleukin is reported to play a central role in regulating inflammation in vasculitis [51]. Conversely, in the context of atherosclerosis, IL-10 alleviates inflammation in the atheroma plaque. Therefore, inadequate IL-10 signaling could diminish the modulation of the inflammatory response in arteries, facilitating the progression of atherosclerotic lesions [52]. In addition, the CSF1R gene encodes a receptor that regulates the growth, differentiation, and function of macrophages. Thus, abnormal expression of this gene could amplify inflammatory responses in blood vessels [53, 54]. Both Medina et al. and Wei et al. found that inhibition of its expression resulted in a decrease in atheroma plaque burden [55, 56].

In the scientific literature, limited availability of information related to the genes under investigation and their association with the two diseases has been observed. Consequently, this analysis will present the studies we have found regarding the biochemistry and signaling pathways linked to these diseases. One of the key players in vascular inflammation and immune response is effector memory T cells (TEM). Activation of the potassium channel Kv1.3 has been observed to play a significant role in their activation. The implication of these mechanisms in atherosclerosis suggests that the regulation of TEM cells and Kv1.3 could be a promising therapeutic strategy for disease management [39]. A relevant genetic polymorphism in the context of atherosclerosis is MTHFR C677T, which has been associated with an increased risk of ischemic stroke and coronary artery disease. This polymorphism is related to a decrease in enzymatic activity and an increase in homocysteine levels, which could contribute to the pathogenesis of atherosclerosis [19]. Likewise, the polymorphisms rs2227631 and rs1799889 of PAI-1 have been identified as potential genetic biomarkers for atherosclerotic diseases [57]. Plasma alpha-aminoadipic acid (2-AAA) has been associated with the development of type 2 diabetes and atherosclerosis. Plasma 2-AAA levels are influenced by common variants in genes related to mitochondrial and macrophage function, and an inverse correlation has been observed between elevated plasma 2-AAA and high-density lipoprotein cholesterol levels [58]. The ApoE4 genotype and metabolic profile also appear to influence coronary artery disease, especially in women. ApoE4 + women with a deficient metabolic profile have a higher risk of subclinical atherosclerosis compared to ApoE4 + women without a deficient metabolic profile and ApoE4- women [59]. VEGFR2 polymorphisms could be used as biomarkers to identify individuals with a high susceptibility to atherosclerotic cardiovascular diseases [60]. Similarly, the D allele in the ACE gene has been associated with an increased risk of atherosclerosis, especially in the European population, and a dose-dependent correlation has been observed [61]. In patients with chronic kidney disease (CKD), the rs495392 polymorphism in the Klotho gene has been associated with decreased odds of atheromatosis progression [62]. Another study in the Chinese population showed that variants rs2241766 and rs266729 are associated with an increased risk of dyslipidemia, atherosclerosis, and coronary artery disease [63]. Importantly, susceptibility to different polymorphisms and disease manifestation vary according to ethnicity [64–68]. Genetic variants in IL-10 have been linked to the development and severity of coronary artery diseases [69]. Furthermore, an autosomal recessive disease called adenosine deaminase 2 deficiency (DADA2) has been recognized, characterized by systemic vascular and inflammatory manifestations, including recurrent strokes [70]. CCL2 levels have been shown to be associated with atherosclerotic disease. CCL2 inhibition shows a positive effect by reducing atherosclerotic lesion size and regulating macrophage and smooth muscle cell accumulation [71]. In another approach, the association of polymorphisms in the BTNL9 gene with atherogenic lipid profiles has been studied, demonstrating the influence of ethnic factors in the genetics of the disease [72, 73]. Recently, it has been discovered that extracellular vesicles (EVs) released by macrophages contain miRNA-503-5p, which participates in atherosclerosis by negatively regulating members of the Smad 1, 2, and 7 family in endothelial and smooth muscle cells, leading to increased inflammation and cell adhesion. This miRNA-503-5p could be considered as a potential therapeutic target [74]. Lastly, the inhibition of ANGPTL3 has shown a cardioprotective effect, and its genetic variation has significant associations with plasma lipidome, suggesting a relevant role in lipid metabolism [75].

One of the prominent findings in this study is the elevated expression of LFA-1 on neutrophils in patients with AAV. The correlation of LFA-1 levels with clinical characteristics of the disease suggests that this adhesion molecule may play a crucial role in the pathogenesis and severity of AAV. Additionally, inhibiting leukocyte-endothelium interaction through LFA-1 and ICAM1 inhibition could be a promising therapeutic strategy to control inflammation in AAV [76]. Another clinically relevant marker is CXCL-13, which shows potential for distinguishing between active vasculitis and long-term remission. Detecting CXCL-13 could be useful for monitoring and managing the disease in AAV patients. Furthermore, the level of MMP-3 is related to renal function, expressed by the estimated glomerular filtration rate (eGFR). Since elevated MMP-3 levels may be associated with renal failure, caution should be exercised when interpreting its elevation in AAV patients with renal dysfunction [77, 78]. Genetic contribution to AAV is also evident in this study. Several genetic variants in both MHC and non-MHC regions have been identified to be associated with AAV. Specifically, HLA-DP shows the strongest association, but significant associations have also been found with genes such as CTLA-4, FCGR2A, PTPN22, SERPINA1, and TLR9. Additionally, different clinical subtypes of AAV may have distinct genetic backgrounds [79]. Regarding molecular mechanisms, B cells and IgG4 appear to be involved in the development and activity of ANCA-associated vasculitis [80]. Furthermore, several studies have demonstrated a correlation between disease activity and the expression of specific miRNAs, suggesting their possible role as biomarkers of disease activity [81, 82]. Type I interferon responses are also implicated in ANCA-associated vasculitis, although genetic studies have not found a direct association [83]. This may indicate a complex interplay between genetic and environmental factors in the pathogenesis of the disease [84–90].

Environmental factors play a crucial role in genetic regulation and protein expression. It has been documented that exposure to pollutants, such as tobacco smoke, particulate matter, and industrial chemicals, increases oxidative stress, which in turn affects the expression of proteins involved in inflammatory and immune responses [91]. Additionally, exposure to ultraviolet radiation and other physical agents can induce damage to genetic material and alter protein expression [92]. In the nutritional realm, numerous antioxidant compounds, such as vitamins, minerals, and polyphenols, have been shown to contribute to the mitigation of oxidative stress and the reduction of inflammation levels. A widely studied case in this context is omega-3 fatty acids [93]. Nutrigenomic studies have shown that compounds such as resveratrol [94, 95], selenium [96], or formulas from traditional Chinese medicine could potentially help improve the pathology [97]. Furthermore, the gut microbiota has been the subject of research concerning vascular diseases, with evidence indicating that metabolites produced by this microbiota have significant effects on health [98]. On the other hand, stress, whether physiological or psychological in nature, induces the release of cortisol and other stress hormones. These mediators promote the production of pro-inflammatory cytokines while simultaneously inhibiting the activity of anti-inflammatory cytokines [99].

According to the findings of our research, the genes CYBB, FCER1G, TYROBP, IL10RA, and CSF1R could represent relevant targets for the study and development of therapeutic strategies aimed at ANCA-associated vasculitis and atherosclerosis. These genes may play a crucial role in the underlying mechanisms of these diseases, and therefore, investigating their function and regulation could offer significant benefits for the development of more effective and specific treatments for these pathological conditions [100–103].

The main limitations of our study lie in the fact that WGCNA and GEO2R can detect correlations but do not establish causality. This implies that the genes identified as commonly expressed between AAV and AS may not be directly responsible for the underlying mechanisms in both conditions. However, as there are no prior studies on this topic, this research may serve as an exploratory investigation into the associations between AAV and AS. While the validation of hub genes and the examination of immune cell infiltration provide valuable insights, additional experimental verification is required to confirm their biological relevance. From this perspective, new gaps in knowledge emerge that warrant further investigation.

## Conclusion

This study reveals that the immune system and inflammation are susceptibility factors for ANCA-associated vasculitis and atherosclerosis. The identified genes (CYBB, FCER1G, TYROBP, IL10RA, and CSF1R) could serve as targets for novel treatments, although further investigations in cells and animal models are required to validate these findings.

## Data Availability

No datasets were generated or analysed during the current study.
